# A 12-year overview of fertility preservation practice in Nordic pediatric oncology centers

**DOI:** 10.1007/s11764-024-01627-x

**Published:** 2024-06-14

**Authors:** Babak Asadi-Azarbaijani, Irma C. Oskam, Kirsi Jahnukainen

**Affiliations:** 1 Faculty of Health Studies, VID Specialized University, PB 184 Vinderen, 0319 Oslo, Norway; 2 The Livestock Production Research Centre, Norwegian University of Life Sciences, Aas, Norway; 3 Department of Pediatrics, University of Helsinki and Helsinki University Hospital, Helsinki, Finland; 4 NORDFERTIL Research Lab Stockholm, Childhood Cancer Research Unit, Karolinska Institute and Karolinska University Hospital, Stockholm, Sweden

**Keywords:** Fertility, Fertility preservation, Children, Cancer, Pediatric, Oncology, Questionnaire

## Abstract

**Purpose:**

Fertility preservation is the only option to safeguard fertility following gonadotoxic treatments. This study aimed to provide an updated status on fertility preservation for pediatric cancer patients in the Nordic countries.

**Methods:**

A questionnaire consisting of 14 questions was sent to directors of 18 main pediatric oncology centers in the Nordic countries in 2010 and 2022. We received information regarding indications, guidelines, counseling, and available fertility preservation options.

**Results:**

The response rates were 89% in 2010 and 72% in 2022. The results reveal an increase in clinical practice guidelines on fertility preservation for cancer patients, from 25% in 2010 to 70% in 2022. Counseling on fertility preservation options in 2022 was more specific and offered to most patients who fulfilled indications for fertility preservation (from 19 to 77%). Sperm cryopreservation continues to be the predominant fertility preservation method for pubertal boys in the Nordic countries. However, there has been a notable increase in the availability of testicular tissue preservation for prepubertal boys (0 to 62%). A similar increase in the offer of ovarian tissue preservation for prepubertal girls (0 to 92%) was observed among pediatric cancer patients.

**Conclusions:**

The past decade has shown commendable advancements in fertility preservation for pediatric cancer patients in the Nordic countries.

**Implications for Cancer Survivors:**

As fertility care evolves globally, continuous assessment of regional practices and challenges is imperative to enhance the quality of care and life for pediatric cancer survivors in the Nordic regions.

**Supplementary Information:**

The online version contains supplementary material available at 10.1007/s11764-024-01627-x.

## Introduction

Fertility preservation for pediatric cancer patients is a central issue among a growing number of childhood and adolescent cancer survivors [1–4]. Various factors, such as type of cancer, age, genetic syndromes, and exposure to cancer treatments, can influence the reproductive system’s functioning in both genders [5]. Infertility due to exposure to gonadotoxic agents is a major factor in the advancement of fertility preservation technologies. Based on the latest international guidelines on fertility preservation, cancer patients scheduled for gonadotoxic treatments should promptly receive referrals for fertility preservation counseling in order to maximize their chances for future parenthood [6, 7].

Since the birth of the first infant conceived in the Nordics via IVF in 1982 (in Gothenburg, Sweden), the Nordic countries have led the charge in the development of assisted reproductive technologies [8]. Their research has significantly contributed to enhancing fertility preservation methods [9]. In 2009, recognizing infertility as a primary concern for post-treatment pediatric cancer patients, pediatric oncologists, and fertility specialists in the Nordic countries established the Nordic Network for Gonadal Preservation after Cancer Treatment in Children and Young Adults. This multidisciplinary partnership led to a consensus statement that the Nordic Society of Pediatric Hematology and Oncology (NOPHO) summarized as Nordic recommendations for fertility preservation in children and adolescents. These were first drafted in 2012 and later revised in 2015 [10, 11]. The recommendations serve as a blueprint for national guidelines in each of the Nordic countries, aiming to integrate fertility preservation and fertility counseling into the clinical routine for pediatric cancer patients and further the development of fertility preservation techniques.

Our study is the first to assess the changes in fertility preservation practices before and after the introduction of the Nordic recommendations for fertility preservation in pediatric oncology centers. The aim is to achieve secure and equal access to fertility preservation options in the Nordic regions.

## Materials and methods

The questionnaire was designed by the authors in order to evaluate the status of fertility preservation practice for pediatric cancer patients in the Nordic countries. It was developed in cooperation with pediatric oncologists and gynecologists who were members of “the Nordic Network for Gonadal Preservation after Cancer Treatment in Children and Young Adults” in 2010 and updated in 2022. The questionnaire consists of 14 multiple choice and open-ended questions. The questions elicited information regarding guidelines, available fertility preservation options, clinical indications, implementation of fertility preservation in practice, and counseling (Appendix 1).

In 2010, in collaboration with “the Nordic Network for Gonadal Preservation after Cancer Treatment in Children and Young Adults,” the questionnaire was sent to directors of 18 main pediatric oncology centers in the Nordic countries. In 2022, another round was sent to the same centers, in association with the Nordic Society of Pediatric Hematology and Oncology (NOPHO). All study centers are public university hospitals with a high volume of pediatric cancer patients. Fertility preservation services for cancer patients in all oncology centers are covered by publicly financed comprehensive healthcare systems.

The questionnaires were answered either by pediatric oncologists alone or in collaboration with other specialists. The directors of each center provided the answers or designated someone in the center to complete the questionnaire. The data collection was conducted electronically via email in 2010 or through a web-based online questionnaire in 2022, with up to three reminders sent. The analysis was carried out anonymously.

### Statistical analysis

Categorical variables as percentage proportions were compared using Fisher’s exact test. The level of statistical significance was set at *P* < 0.05. All calculations were performed using the IBM SPSS statistical software, version 28.

## Results

In total, 16 oncology centers responded to the questionnaire in 2010, and 13 centers did so in 2022 (Table 1). The response rates were 89% in 2010 and 72% in 2022. A breakdown of the responding centers by country is presented in Table 1. The comparison of results between 2010 and 2022 is between all Nordic countries, except Iceland in 2010 (Table 1).

**Table 1:** The number of responding centers by country

Country	Year 2010	Year 2022
Denmark	4/4	2/4
Finland	4/4	3/4
Iceland	0/1	1/1
Norway	3/4	3/4
Sweden	5/5	4/5
Total	16/18	13/18

### Counseling on fertility preservation options

At most centers, pediatric oncologists were primarily responsible for providing counseling on fertility preservation options, accounting for 88% in 2010 and 100% in 2022. Counseling on fertility preservation options in 2010 was offered the most to all cancer patients (63%) whereas counseling in 2022 was more specific and offered primarily to patients who fulfilled indications for fertility preservation (77%). In some centers, counseling on fertility preservation options was a collaboration between oncologists and other specialists. Only a few centers offered counseling on fertility preservation options by oncology nurses, endocrinologists, andrologists, or gynecologists.

### Clinical practices and guidelines for fertility preservation

In 2010, 25% of centers had established national guidelines for fertility preservation within their respective countries [12]. By 2022, the percentage had notably increased to 77%. Additionally, one center reported having developed local guidelines specific to their center (8%) in 2022. This indicates a significant increase in the establishment of national guidelines over the 12-year period (*P* value < 0.05) (Appendix 1).

The response rate for the question “Do you have guidelines for fertility preservation by therapeutic agents, age of patient, or other criteria?” was 31% in 2010, and it increased to 69% in 2022 (Appendix 2). Although there was variation in the responses, the majority reported that fertility preservation would be performed before initiating high gonadotoxic treatments, including hematopoietic stem cell transplantation (HSCT), irradiation exposing gonads, and high-dose alkylators. Additionally, the primary emphasis was on sexual maturity when selecting fertility preservation options. Age, gender, and diagnosis for the last reported cases that were offered fertility preservation were in line with the respondents’ answers in this study (Appendix 2).

In response to the question “Do you ever offer sperm/testicular tissue/ovarian cortical tissue/oocyte preservation to individuals with the following diagnosis?,” answers varied among all centers. However, there was an increase in the availability of fertility preservation options for a broader range of cancer diagnoses in 2022 (Table 2). Between 2010 and 2022, there was only a significant increase in the number of centers offering fertility preservation options for patients diagnosed with non-Hodgkin lymphoma and Wilms tumor (Table 2).

**Table 2:** Proportion of centers offering sperm, testicular tissue, ovarian cortical tissue, or oocyte preservation by cancer diagnosis in 2010 and 2022

Diagnosis	2010 No. of centers/total (%)	2022 No. of centers/total (%)	Difference %
Hodgkin’s disease	12/16 (75)	11/13 (85)	+ 10
Non-Hodgkin lymphoma	9/16 (56)	12/13 (92)	+ 36*
Acute lymphoblastic leukemia	6/16 (38)	8/13 (62)	+ 24
Acute myeloid leukemia	4/16 (25)	8/13 (62)	+ 37
Wilms tumor	1/16 (6)	6/13 (46)	+ 40*
Ewing/soft tissue sarcoma	9/16 (56)	11/13 (85)	+ 29
Osteosarcoma	7/16 (44)	10/13 (77)	+ 33
CNS tumors	3/16 (19)	5/13 (39)	+ 20
Germ cell tumors	4/16 (25)	6/13 (46)	+ 21
Before stem cell transplantation	11/16 (69)	12/13 (92)	+ 23

*P value < 0.05

### Male fertility preservation

**Fig. 1:**
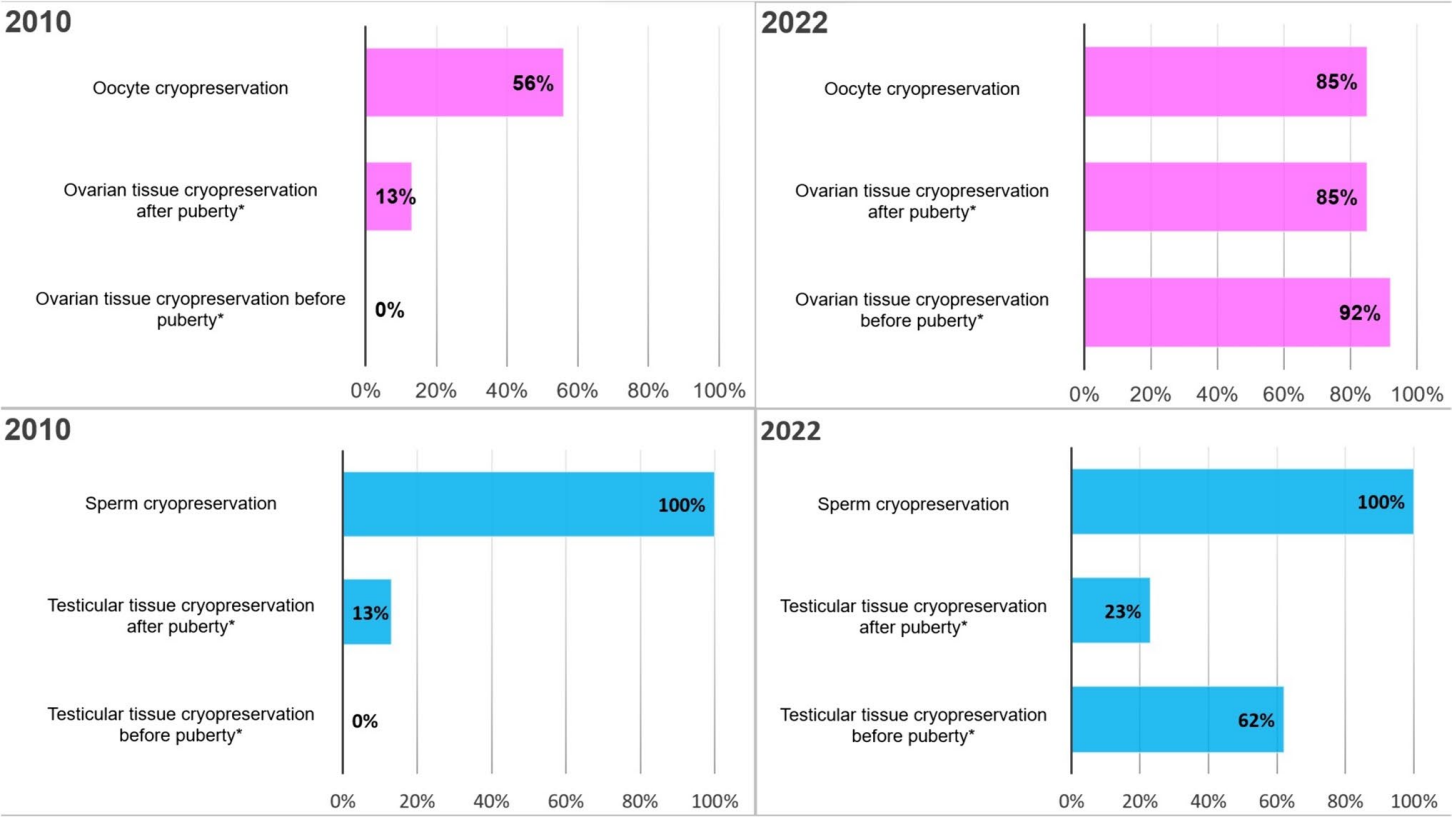
The most relevant options for male and female fertility preservation offered in the Nordic countries. An asterisk (*) indicates a *P* value < 0.05

Figure 1 highlights the most relevant options for male and female fertility preservation offered to cancer patients in the Nordic countries. The results indicate that the sexual maturity of boys was the focus when considering sperm cryopreservation. A significant increase in the activity of cryopreserving prepuberal testicular tissue was observed between 2010 and 2022 (from 0% in 2010 to 62% in 2022) (Fig. 1). However, Norway has not yet established the practice of cryopreserving testicular tissue for prepubertal boys. In 2022, 46% of centers reported that they offer male fertility preservation options after treatment to some patients. In 2010, there were no reported male fertility preservation options offered after cancer treatment (Appendix 1).

For postpubertal boys, there was a consistently high rate of sperm banking offered across all centers in both 2010 and 2022, standing at 100% for both years. The sexual maturity of boys for sperm production in both 2010 and 2022 was decided by consultation with patients/parents, clinical examination according to Tanner stage, and an assessment of testicular development. In 2010, some respondents also mentioned collecting morning urine samples and collaborating with pediatric endocrinologists. Most respondents in both 2010 and 2022 reported that sperm samples were obtained at the sperm laboratory (88% and 77%, respectively). Conversely, in 2010 and 2022, some respondents reported that sperm samples were obtained at the patient’s home or hospital room (56% and 31%, respectively) (Appendix 1).

In most centers, pediatric oncologists were responsible for requesting sperm sample collection. In some centers, this was also requested by endocrinologists, oncology nurses, or andrologists. In one center in 2022, andrologists held the main responsibility for requesting sperm collection, accounting for 8% (Appendix 1).

Oncology centers had different responses regarding methods for collecting sperm when pubertal boys were unable to produce sperm naturally (Table 3). The results indicate a significant percentage increase in the use of electroejaculation for producing sperm samples from such boys (Table 3). There was also a slight increase in preserving testicular tissue for pubertal boys, from 6% in 2010 to 15% in 2022. Some centers still have no fertility preservation options for pubertal boys who are unable to produce sperm naturally (Table 3).

**Table 3:** Options for sperm collection in pubertal boys who were unable to produce sperm naturally

Fertility preservation options	2010 No. of centers/total (%)	2022 No. of centers/total (%)	Difference %
Testicular tissue preservation	1/16 (6)	2/13 (15)	+ 9
PESA^a^/TESA^b^	1/16 (6)	1/13 (8)	+ 2
Electroejaculation	3/16 (19)	8/13 (62)	+ 43*
Try again ejaculation	5/16 (31)	2/13 (15)	-16
No options	7/16 (44)	5/13 (39)	-5

^a^Percutaneous epidydimal sperm aspiration (PESA), ^b^Testicular sperm aspiration (TESA)

*P value < 0.05

### Female fertility preservation

The cryopreservation of ovarian tissue significantly increased for both prepubertal and pubertal girls in 2022 (Fig. 1). Iceland, however, has not established the practice of ovarian tissue cryopreservation. Furthermore, there was an increase in oocyte cryopreservation for pubertal girls, from 56% in 2010 to 85% in 2022. The response to the question, “Do you have any routines for treating girls who have recovered from ovarian failure?” indicates a significant improvement in access to fertility treatments after cancer therapy for female cancer survivors at risk of ovarian failure between 2010 and 2022. In 2010, only 6% of respondents reported having routines for such treatments, whereas this figure notably increased to 62% by 2022 (Appendix 1). After treatment, the girls were followed up by gynecologists and/or endocrinologists.

### Collaboration with services for fertility preservation

In 2010, 50% of the responding centers had an established collaboration with services specializing in ovarian tissue cryopreservation, 19% with services offering ovarian stimulation and oocyte cryopreservation, and 94% with services for sperm collection and preservation. In 2022, these numbers changed to 85% for ovarian tissue cryopreservation, 69% for ovarian stimulation and oocyte cryopreservation, and 92% for sperm collection and preservation. No centers reported collaboration with services for testicular tissue cryopreservation in 2010. However, by 2022, 69% of centers had established such collaboration (Appendix 1).

## Discussion

The significance of fertility preservation for pediatric cancer patients has been increasingly recognized, not only globally but also within the Nordic regions, particularly given the growing population of childhood and adolescent cancer survivors [13, 14]. This surge in awareness is largely due to the potential adverse effects of gonadotoxic treatments on fertility [5]. In the Nordic countries, fertility preservation is considered an essential component of cancer care, with comprehensive healthcare systems, public funding, and strong support networks ensuring that individuals facing cancer treatment have access to fertility preservation options and support services [15].

### Counseling on fertility preservation options

This study’s findings highlight changes in counseling practices. In the last 12 years, counseling has become more specialized, focusing on those patients who fulfill specific indications for fertility preservation, pointing to a more tailored and effective approach. This shows a progression in the clinical practice routine for offering counseling on fertility preservation options. The finding indicates improved fertility preservation care and strategies among pediatric oncologists, gynecologists, oncology nurses, and fertility treatment providers [16]. Fertility counseling may also increase awareness of fertility preservation options among pediatric cancer patients and parents [16]. However, there are still some centers that do not offer counseling on fertility preservation options to relevant patients, potentially affecting the quality of fertility care among pediatric cancer patients.

### Clinical practices and guidelines for fertility preservation

By assessing the fertility preservation practices within Nordic oncology centers over a period of 12 years, the study illuminates significant advancements in this vital area of patient care and highlights the challenges faced in harmonizing cancer treatment plans across the Nordic pediatric oncology centers.

The data reveal a clear progression in the fertility preservation methods and guidelines within this period. By 2022, substantial progress in the establishment of national guidelines for fertility preservation was observed, aligning with the global attention on fertility care for pediatric cancer survivors. This is a clear indication of the role that Nordic recommendations and practices have had on fertility preservation protocols, as well as their subsequent incorporation into clinical routines for pediatric cancer patients in the Nordic countries. However, disparities in the implementation of and adherence to these guidelines among Nordic oncology centers point to potential barriers, such as the lack of structured and coordinated fertility preservation, limited guideline knowledge, and inconsistent adherence by oncologists and other healthcare providers [17–19]. Research also shows that existing clinical practice guidelines for fertility preservation that were developed by different institutions vary substantially, and only about one-third of the identified guidelines were found to be of sufficient quality [7]. This may reduce the ability of oncologists and other healthcare providers to deliver high-quality fertility care to pediatric cancer patients.

One of the most significant insights from the study is the increase in the offering of fertility preservation options across different diagnoses, especially for patients diagnosed with non-Hodgkin’s lymphoma and Wilms tumor. This serves to emphasize the role of effective collaboration between oncologists and fertility providers, together with common guidelines, in enhancing the identification of children at increased risk of subfertility caused by various cancers and their treatments.

### Male fertility preservation

For boys, there is a growing emphasis in the Nordic oncology centers on evaluating sexual maturity before opting for sperm cryopreservation. Over the past decade, fertility preservation methods for postpubertal boys have already become a part of clinical practice and offered routinely to all patients who fulfill indications for fertility preservation [6, 20]. The responses to the questionnaire indicate that electroejaculation has been selected more than the other options by specialists in the Nordic countries. This is consistent with current studies that recommend considering electroejaculation as a useful option for sperm cryopreservation [20]. However, current studies have not shown any evidence regarding pregnancies or live births after sperm cryopreservation via electroejaculation in pediatric cancer patients [20].

Many countries—including the Nordics—offer testicular tissue cryopreservation for fertility preservation in prepubertal boys undergoing gonadotoxic treatments [21]. Thus far, this procedure is experimental, and there are no reports on pregnancy or live births after using frozen human testicular tissue [21]. The international guidelines recommend testicular tissue cryopreservation for prepubertal boys only within the context of an approved research protocol [20]. Despite the experimental aspect of the procedure, families and patients have shown willingness to preserve testicular tissue for the future [22]. In 2022, more centers reported that they offer male fertility preservation following cancer treatment. This practice aligns with Nordic guidelines, which do not mandate testicular biopsy prior to initiating gonadotoxic treatment. Correspondingly, a recent comprehensive international report revealed that approximately 30% of patients underwent biopsies after receiving treatments with low to medium gonadotoxic risk [23]. While male fertility preservation practice is broadly similar across the Nordic centers, there remain variations. International collaboration between the centers offering this experimental procedure will be important for ensuring good outcomes for young males at risk of future infertility.

### Female fertility preservation

In terms of gender-specific findings, female fertility preservation witnessed a dramatic rise in the cryopreservation of ovarian tissue, both for prepubertal and postpubertal girls, mirroring global advancements in female fertility preservation techniques [24, 25]. The increasing reliance on techniques such as ovarian tissue cryopreservation, especially for prepubertal cancer patients, reinforces the progress in this domain [25]. This is in line with the female fertility preservation options offered in the Nordic countries over the last decade. The evidence regarding the success of this intervention is largely based on case reports and case series and may still be deemed experimental by some centers [26]. However, with increasing evidence of live births and the return of endocrine function, the international guidelines recommend considering ovarian tissue cryopreservation for prepubertal girls [6, 27, 28].

Oocyte cryopreservation stands as a clinically established method for pubertal girls [26]. This method has undergone significant global refinement and is now routinely in use [26, 27]. In the Nordic countries, the provision of oocyte cryopreservation has increased over the last 12 years. However, this option is currently only extended to postmenarcheal cancer patients if their prognosis would not be compromised by a delay in treatment initiation [27].

Over the past decade, there has been a heightened emphasis on preserving the fertility of pediatric female cancer patients after cancer therapy in the Nordic countries. Consequently, there is a need for endocrinologists and gynecologists to continue monitoring girls who are at risk of ovarian failure following cancer treatment [29]. The findings from this study indicate that several Nordic oncology centers have implemented protocols for managing girls at risk of ovarian failure after cancer treatment. This monitoring should encompass reproductive health assessments, including evaluations of ovarian function, planning for future pregnancies, and the initiation of hormone replacement therapy as necessary [30, 31]. Such measures are poised to enhance the quality of life for female cancer survivors.

### Collaboration with services for fertility preservation

In the Nordic countries, fertility preservation is limited to fertility preservation programs at academic reproductive centers that belong to major university hospitals [29]. Consequently, there has been a growing need for collaboration among oncology centers to establish centralized biobanks in each country. This initiative has provided the opportunity to safeguard fertility potential, thereby potentially enhancing pediatric cancer survivors’ quality of life.

Furthermore, collaboration between the Nordic centers and fertility preservation services has grown substantially, emphasizing the integrated approach that Nordic centers have adopted over the years. This collaboration remains important, to develop transparent and rigorous clinical practice guidelines that can optimize fertility preservation for pediatric cancer patients in all centers. This might balance the harms and benefits of methods for fertility preservation in different risk groups.

The present study possesses certain limitations. Firstly, there is a difference between some of the oncology centers that responded in 2010 and those that responded in 2022. Additionally, respondents failed to address all open-ended questions. The lack of response implies limitations on information from all centers. Moreover, since the questionnaire was in English and not in the respondents’ native languages, language barriers may have led to variations in understanding the questions across different countries and time periods. Furthermore, it is also not feasible to estimate the percentage of all pediatric cancer patients treated at the responding centers.

Despite these limitations, the study’s findings demonstrate that collaboration among Nordic oncology centers has facilitated the establishment of national guidelines in each country. This collaboration has notably enhanced the provision of fertility preservation counseling and services, thereby promoting the integration of oncology and fertility care.

## Conclusion

While the past decade has shown commendable advancements in fertility preservation for pediatric cancer patients in the Nordic countries, challenges persist. As fertility care evolves globally, continuous assessment of regional practices and challenges is imperative to enhance the quality of care and life for pediatric cancer survivors in the Nordic regions.

## Supplementary Information

Below is the link to the electronic supplementary material.

Supplementary file1 (DOCX 36 KB)

Supplementary file2 (DOCX 29 KB)

## Data Availability

No datasets were generated or analysed during the current study.
