# Supplementary material for: A 12-year overview of fertility preservation practice in Nordic pediatric oncology centers
Source: J Cancer Surviv. 2024 Jun 14;20(1):12–9. doi: 10.1007/s11764-024-01627-x (PMC12906589; doi:10.1007/s11764-024-01627-x)
Supplement: Supplementary file 1 — Supplementary file1 (DOCX 36 KB) [file 11764_2024_1627_MOESM1_ESM.docx]

**Appendix 1**

**Questionnaire on fertility preservation practices among pediatric cancer patients in the Nordic countries for the year 2010 and 2022.**

| **Question** | **2010**  **No. of centers/total (%)** | **2022**  **No. of centers/total (%)** |
| --- | --- | --- |
| **Legislation/Guideline** | | |
| Are there any national guidelines on fertility preservation for cancer patients? | | |
| Yes | 4/16 (25) | 10/13 (77) |
| No | 12/16 (75) | 2/13 (15) |
| Local guidelines are available. | - | 1/13 (8) |
| It will be provided | - | - |
| Do not know | - | - |
| Choose the male fertility preservation options offered in your country: | | |
| Testicular tissue cryopreservation before puberty | - | 8/13 (62) |
| Testicular tissue cryopreservation after puberty | 2/16 (13) | 3/13 (23) |
| Sperm cryopreservation | 16/16 (100) | 13/13 (100) |
| Others please specify: | - | - |
| Choose the female fertility preservation options offered in your country: | | |
| Ovarian tissue cryopreservation before puberty | - | 12/13 (92) |
| Ovarian tissue cryopreservation after puberty | 2/16 (13) | 11/13 (85) |
| Oocyte cryopreservation | 9/16 (56) | 11/13 (85) |
| Others please specify: | - | - |
| Does your center have guidelines available regarding: | | |
| Sperm collection and preservation? | 16/16 (100) | 13/13 (100) |
| Testicular tissue collection and preservation? | 2/16 (13) | 8/13 (62) |
| Ovarian cortical tissue collection and preservation? | 2/16 (13) | 11/13 (85) |
| Oocyte cryopreservation? | 9/16 (56) | 11/13 (85) |
| If yes, do you have guidelines for fertility preservation by therapeutic agents, age of patient, or other criteria? If so, which?   1. Therapeutic agents (e.g. “ovarian cortical tissue preservation is offered before stem cell transplantation if a girl has had menarche”) 2. Age of patient (e.g.” sperm preservation is offered to boys older than 15 years”) 3. Other criteria | See appendix 2 for full response | |
| **Practice** | | |
| Does your center have an established collaboration with service for: | | |
| Sperm collection and preservation? | 15/16 (94) | 12/13 (92) |
| Testicular tissue collection and preservation? | - | 9/13 (69) |
| Ovarian cortical tissue collection and preservation? | 8/16 (50) | 11/13 (85) |
| Ovarian stimulation and oocyte cryopreservation? | 3/16 (19) | 9/13 (69) |
| Do you ever offer sperm/testicular tissue/ovarian cortical tissue/oocyte preservation to individuals with the following diagnosis? | | |
| Hodgkin’s disease | 12/16 (75) | 11/13 (85) |
| Non-Hodgkin’s lymphoma | 9/16 (56) | 12/13 (92) |
| Acute lymphoblastic leukaemia at diagnosis | 6/16 (38) | 8/13 (62) |
| Acute myeloid leukaemia at diagnosis | 4/16 (25) | 8/13 (62) |
| Wilm’s tumors | 1/16 (6) | 6/13 (46) |
| Ewing’s/ soft tissue sarcoma | 9/16 (56) | 11/13 (85) |
| Osteosarcoma | 7/16 (44) | 10/13 (77) |
| CNS tumors | 3/16 (19) | 5/13 (39) |
| Germ cell tumors | 4/16 (25) | 6/13 (46) |
| Before stem cell transplantation | 11/16 (69) | 12/13 (92) |
| Do you remember the age, gender, and diagnosis of the most recent patient you offered sperm/ testicular tissue/ ovarian cortical tissue/ oocyte preservation? | See appendix 2 for full response | |
| Do you offer counseling on fertility preservation options for patients and parents? | | |
| Yes, all patients and parents | 10/16 (63) | 1/13 (6) |
| Yes, some | 1/16 (6) | 2/13 (15) |
| Yes, only those who fulfill indications for fertility preservation | 3/16 (19) | 10/13 (77) |
| No | 2/16 (13) | 0 |
| I do not know | 0 | 0 |
| Who is responsible for fertility consultation? | | |
| Oncologist | 14/16 (88) | 13/13 (100) |
| Oncology nurse | 1/16 (6) | 2/13 (15) |
| Gynecologist | 4/16 (25) | 8/13 (62) |
| Andrologist | 2/16 (13) | 0 |
| Endocrinologist | 5/16 (31) | 3/13 (23) |
| Others | 0 | 0 |
| There is no consultation | 0 | 0 |
| **Male fertility preservation** | | |
| Who is asking for sperm collection? | | |
| Oncologist | 15/16 (94) | 12/13 (92) |
| Oncology nurse | 1/16 (6) | 2/13 (15) |
| Gynecologist | 3/16 (19) | 0 |
| Andrologist | 1/16 (6) | 1/13 (8) |
| Endocrinologist | 4/16 (25) | 2/13 (15) |
| Others | 0 | 0 |
| How do you decide that a boy is old enough to produce a sperm sample? | | |
| Puberty stage | 11/16 (79) | 7/13 (54) |
| Ask the patients | 5/16 (31) | 6/13 (46) |
| Others | 0 | 0 |
| Where is the sperm sample produced? | | |
| At the sperm laboratory | 14/16 (88) | 10/13 (77) |
| At the patient’s home | 5/16 (31) | 4/13 (31) |
| In the patient’s hospital room | 9/16 (56) | 4/13 (31) |
| Others | 0 | 0 |
| What do you do if a boy is unable to produce a sperm sample? | | |
| Testicular tissue preservation | 1/16 (6) | 2/13 (15) |
| PESA^a^/TESA^b^ | 1/16 (6) | 1/13 (8) |
| Electroejaculation | 3/16 (19) | 8/13 (62) |
| Try again ejaculation | 5/16 (31) | 2/13 (15) |
| No options | 7/16 (44) | 5/13 (39) |
| Do you ever offer sperm/ testicular tissue preservation after the cancer treatment? | | |
| Yes, for all | 0 | 0 |
| Yes, some patients | 0 | 6/13 (46) |
| No | 0 | 7/13 (54) |
| Do not know | 0 | 0 |
| **Female fertility preservation** | | |
| Do you have any routines for treating girls who have recovered from ovarian failure? | | |
| Consultation with gynecologist | 1/16 (6) | 8/13 (62) |
| Consultation with endocrinologist | 1/16 (6) | 8/13 (62) |
| No routine | 15/16 (94) | 5/13 (38) |
| Others | 0 | 0 |

1. Percutaneous epidydimal sperm aspiration (PESA), b. Testicular sperm aspiration (TESA)

---------------------------------------------------------------------------------------------------------------------------

**Title:** A twelve-year overview of fertility preservation practice in the Nordic pediatric oncology centers

Journal of Cancer Survivorship

Authors: Babak Asadi-Azarbaijani^1^; Irma C Oskam^2^; Kirsi Jahnukainen^3,4^

1. Faculty of Health Studies, VID Specialized University, Oslo, Norway
2. The Livestock Production Research Centre, Norwegian University of Life Sciences, Aas, Norway
3. Department of Pediatrics, University of Helsinki and Helsinki University Hospital, Helsinki, Finland
4. NORDFERTIL Research Lab Stockholm, Childhood Cancer Research Unit, Karolinska Institute and Karolinska University Hospital, Stockholm, Sweden

Corresponding author: Babak Asadi-Azarbaijani; [babak.asadi@hotmail.com](mailto:babak.asadi@vid.no)
