# Supplementary material for: A 12-year overview of fertility preservation practice in Nordic pediatric oncology centers
Source: J Cancer Surviv. 2024 Jun 14;20(1):12–9. doi: 10.1007/s11764-024-01627-x (PMC12906589; doi:10.1007/s11764-024-01627-x)
Supplement: Supplementary file 2 — Supplementary file2 (DOCX 29 KB) [file 11764_2024_1627_MOESM2_ESM.docx]

**Appendix 2**

The response to questions “Do you have guidelines for fertility preservation by therapeutic agents, age of patient, or other criteria?” and “Do you remember the age, gender, and diagnosis of the most recent patient you offered sperm/ testicular tissue/ ovarian cortical tissue/ oocyte preservation?”.

| **Country** | **2010** | **2022** | **2010** | **2022** | **2010** | **2022** | **2010** | **2022** |
| --- | --- | --- | --- | --- | --- | --- | --- | --- |
|  | **Therapeutic agents** | | **Age** | | **Others** | | **Recent case** | |
| Denmark | NA | OTC before HSCT and high dose Ifosfamide | NA | SC for pubertal boys | NA | NA | NA | Boy 17y, Acute myeloid leukemia |
| Denmark | NA | - | NA | - | NA | - | NA | - |
| Denmark | HDC, HSCT | NA | NA | SC for pubertal boys | NA | NA | Girl 10y, Ewing | NA |
| Denmark | NA | - | NA | - | NA | - | NA | - |
| Finland | NA | - | NA | - | NA | - | NA | - |
| Finland | NA | Before HSCT or gonadal irradiation or high dose alkylators | Boys> 13y | Girls <16y OTC for research, Boys at sextual maturity stage 3 or higher are offered SC and girls >16 OC | NA | Hematological malignancies are discussed | Boy 16y Hodgkin | Girl 18y, Leukemia  Boy 16y, HSCT |
| Finland | NA | NA | Boys> 13y | NA | NA | NA | Boy 14y, Acute lymphoblastic leukemia | NA |
| Finland | NA | OC beside OTC before HSCT & ovarian irradiation. SC before any therapy | NA | Girls & boys after puberty | NA | Girls OC beside OTC before HSCT & ovarian irradiation. SC for pubertal boys before any therapy | NA | Girls 3y HSCT |
| Iceland | NA | NA | NA | Judged by maturation | NA | Boys if over 90% chance of infertility. No protocol for girls. | NA | NA |
| Norway | Before HSCT | According to treatment | Boys> 13y | After puberty | NA | NA | NA | NA |
| Norway | NA | NA | NA | NA | NA | International guideline + Oslo guideline for freezing of tissues | Girl 15y, Osteosarcoma | Male 15y, Acute myeloid leukemia |
| Norway | Before HSCT Alkylating agents | - | Boys>13y & girls>10y | - | NA | - | Boy 14y Hodgkin | - |
| Norway | - | OTC for Girls alkylating dose (CED >6 g/m2) and/or radiation to the ovaries. Boys, SC for high alkylating agents dose (CED >4 g/m2) and or radiation involving the testis. | - | No age limitation for girls. Boys after puberty for SC | - | No individual decision. Not leukemias | - | Boy 15y Ewing |
| Sweden | Before BMT alkylators | NA | Boys> 13y | NA | NA | NA | Boy 15y Leukemia | NA |
| Sweden | NA | High risk of infertility (undergo HDC/HSCT) | NA | TTC most for all prepubertal | NA | NA | NA | Boy 15y Hodgkin |
| Sweden | NA | NA | NA | NA | NA | NA | NA | NA |
| Sweden | NA | According to protocol | Pubertal boys | Depends on pubertal state. | NA | NA | Boy 15y, BMT | Boy 14y Osteosarcoma |
| Sweden | Boys and Girls before BMT only Leukemia | - | Age >10y | - | Stabile hematological status | - | Boy 17y before BMT | - |

Not Available (NA), Ovarian cryopreservation (OC), Ovarian tissue cryopreservation (OTC), Sperm cryopreservation (SC), Testicle tissue cryopreservation (TTC) High-Dose Chemotherapy (HDC), Hematopoietic Stem Cell Transplantation (HSCT), Bone Marrow Transplantation (BMT)

-----------------------------------------------------------------------------------------------------------------------------------------------------------------------------------------------------------------------------------------

**Title:** A twelve-year overview of fertility preservation practice in the Nordic pediatric oncology centers

Journal of Cancer Survivorship

Authors: Babak Asadi-Azarbaijani^1^; Irma C Oskam^2^; Kirsi Jahnukainen^3,4^

1. Faculty of Health Studies, VID Specialized University, Oslo, Norway
2. The Livestock Production Research Centre, Norwegian University of Life Sciences, Aas, Norway
3. Department of Pediatrics, University of Helsinki and Helsinki University Hospital, Helsinki, Finland
4. NORDFERTIL Research Lab Stockholm, Childhood Cancer Research Unit, Karolinska Institute and Karolinska University Hospital, Stockholm, Sweden

Corresponding author: Babak Asadi-Azarbaijani; [babak.asadi@hotmail.com](mailto:babak.asadi@vid.no)
